# Saffold Virus Infection in Children, Malaysia, 2009

**DOI:** 10.3201/eid1708.101380

**Published:** 2011-08

**Authors:** Kaw Bing Chua, Kenny Voon, Meng Yu, Wan Nor Azlina Wan Ali, Abdul Rasid Kasri, Lin-Fa Wang

**Affiliations:** Author affiliations: National Public Health Laboratory, Sungai Buloh, Selangor, Malaysia (K.B. Chua, W.N.A.W. Ali, A.R. Kasri);; International Medical University, Bukit Jalil, Kuala Lumpur, Malaysia (K.B. Chua, K. Voon);; Australian Animal Health Laboratory, Geelong, Victoria, Australia (M. Yu, L.-F. Wang)

**Keywords:** picornavirus, cardiovirus, Saffold virus, serologic surveillance, viruses, children, Malaysia, letter

**To the Editor:** Since 2007, a new cardiovirus, named Saffold virus (SAFV), has been isolated from human specimens in the United States, Canada, the Netherlands, and People’s Republic of China (*1**–**4*). Concurrent investigations also showed that SAFV could be detected in feces and respiratory secretions of children in other countries, and genetic analysis showed the circulation of different genetic lineages of SAFVs in various parts of the world. This new virus belongs to the genus *Cardiovirus*, in the family *Picornaviridae* (*5*). Here we report isolation of a new SAFV in Malaysia, designated SAFV-Penang to reflect the locality of isolation in Malaysia.

A 5-year-old girl was brought to a government outpatient clinic on November 18, 2009, with reported fever and sore throat for 3 days. The fever was described as of high grade with occasional episodes of rigor, accompanied by profuse sweating and myalgia, lethargy, and loss of appetite. The child had nasal blockage, mild runny nose, and dry cough. She vomited twice on day 3 of illness and had abdominal pain but no diarrhea. There was no history of similar illness affecting other family members, who lived in a semirural area within the state of Penang, Malaysia. Acute pharyngitis/acute influenza-like illness was provisionally diagnosed, and a throat swab specimen was collected in virus transport medium for virus isolation by using established procedures (*6*).

The throat swab sample was treated with antimicrobial drugs for 1 h before the cells were added to MDCK, Vero, and Hep-2 cells. On the fifth day postinoculation (dpi), a lytic form of cytopathic effect (CPE), similar to the type of CPE from enterovirus infection, was noted in Hep-2 but not in Vero or MDCK cells. The progress of CPE was slow, and full CPE was achieved on 9 dpi. On 8 dpi, a 0.5-mL aliquot containing infected Hep-2 cell suspension was removed and processed for indirect immunofluorescence assay by using a panel of commercial typing monoclonal antibodies for human enteroviruses. The infected Hep-2 cells reacted strongly with broad reactive pan-enterovirus monoclonal antibodies (catalog no. 3360, Chemicon Inc., Temecula, CA, USA) but failed to react with any type-specific monoclonal antibodies (data not shown).

After 3 passages in Hep-2 cells, culture supernatant was subsequently passed into Vero cells. After an additional 3 passages, the virus was fully adapted to grow in Vero cells and was able to induce visible CPE 1 dpi and full CPE by 4 dpi.

Partial genome sequence of the virus was initially obtained by using a random priming and amplification method as described (*7*). Full-length sequence was then determined by using primers designed according to the partial genome sequences of SAFV-Penang and genome sequences of other SAFV strains available in GenBank (primer sequences are available on request). The viral genome of SAFV-Penang is 8,073 nt (full sequence deposited in GenBank under accession no. HQ162476). As for other SAFVs, a single long open reading frame of 2,295 aa was detected. Phylogenetic analysis based on the complete amino acid sequences of the polyproteins of different SAFVs indicated that SAFV-Penang is a type 3 SAFV (*8*). It is most closely related to the SAFV-3 isolated in the Netherlands in 2007.

To determine the prevalence of SAFV infection among children in Malaysia, we conducted a seroprevalence study by using 400 serum samples collected during 2009 from children 10–12 years of age under the national hepatitis B postvaccination serosurvey. The serum panel comprised 80 samples containing equal numbers of boys and girls from each of the 5 states: Penang (northwestern), Selangor (central-western), and Kelantan (northeastern) of peninsular Malaysia, and Sabah and Sarawak of eastern Malaysia in Borneo Island. Screening for SAFV-specific antibodies was conducted by using an indirect immunofluorescence antibody test on SAFV-infected Vero cells as described (*9*). The results (Table) indicated that >70% of schoolchildren surveyed had been exposed to the virus. The seropositive rate ranged from 67.5% (Penang) to 75.0% (Kelantan and Sabah), with no significant difference among different parts of the country (χ^2^ = 1.60, df = 4, p = 0.8091). The seropositive rate did not differ significantly by sex (χ^2^ = 0.32, p = 0.5734).

In summary, a new SAFV was discovered in Malaysia by direct virus isolation during an investigation of a febrile patient. Subsequent serologic study indicated that a high percentage of children 10–12 years of age had been exposed to this virus. Further study is required to determine the public health implications of SAFV infection in Southeast Asia, especially in cases in which co-infection with other pathogens might potentially lead to different clinical outcomes.

**Table:** Prevalence of SAFV-specific IgG in serum from children 10–12 years of age, by sex, Malaysia, 2009*

State of residence	No. children	No. positive serum samples†			
		+	++	+++	Total no. (%)
Penang					
M	40	21	7	0	28 (70.0)
F	40	19	5	2	26 (65.0)
Total	80	40	12	2	54 (67.5)
Kelantan					
M	40	16	8	5	29 (72.5)
F	40	18	11	2	31 (77.5)
Total	80	34	19	7	60 (75.0)
Selangor					
M	40	16	11	5	32 (80.0)
F	40	22	5	0	27 (67.5)
Total	80	38	16	5	59 (73.8)
Sabah					
M	40	16	9	4	29 (72.5)
F	40	24	6	1	31 (77.5)
Total	80	40	15	5	60 (75.0)
Sarawak					
M	40	22	6	3	31 (77.5)
F	40	19	5	4	28 (70.0)
Total	80	41	11	7	59 (73.8)

*SAFV, Saffold virus; Ig, immunoglobulin †The degree of positive reactivity was classified into 3 groups: weak (titer <0–40), medium (titer 80–160), and strong (titer 320–1,280) according to the staining intensity on SAFV-infected Vero cells (data not shown). The classification was further confirmed by correlation with virus neutralizing titer from 20 samples in each group, with + samples having a titer of 0–40, ++ samples 80–160, and +++ samples 320–1,280, respectively.
